# Current status and future directions of amyotrophic lateral sclerosis research in Africa: a perspective

**DOI:** 10.1097/MS9.0000000000000814

**Published:** 2023-05-10

**Authors:** Nicholas Aderinto, Opeyemi Muili AbdulBasit, Oluwatosin Afolayan, Mubarak Mustapha Jolayemi

**Affiliations:** a Department of Medicine and Surgery, Ladoke Akintola University of Technology, Ogbomoso; b Faculty of Basic Medical Sciences, University of Ilorin, Ilorin, Nigeria

**Keywords:** Africa, amyotrophic lateral sclerosis, neurodegenerative disease, research

## Abstract

Amyotrophic lateral sclerosis (ALS) is a devastating neurodegenerative disease with a global burden. Despite significant strides in ALS research, challenges facing ALS research in Africa are substantial. This paper discusses the current status and future directions of ALS research in Africa. Challenges and opportunities for ALS research in the region are highlighted, including limited funding and resources, the need for collaboration, and capacity building. Emerging technologies in ALS research in Africa are described, including telemedicine, neuroimaging, and genetic studies. Priorities for future ALS research in Africa are identified, including epidemiological studies, developing culturally appropriate diagnostic and management tools, and clinical trials of emerging treatments. Addressing these priorities will be critical to advancing ALS research and improving patient outcomes in Africa.

## Introduction

Highlights

- Limited resources hinder amyotrophic lateral sclerosis (ALS) research in Africa, resulting in a lack of data and treatment options.
- Collaborative efforts between African governments, research institutions, and international organisations are needed to support ALS research in Africa.
- Promising research directions include the study of risk factors, biomarkers, and novel treatments, which could lead to significant advancements in ALS research and treatment in Africa.

Amyotrophic lateral sclerosis (ALS), also known as Lou Gehrig’s disease, is a rare and fatal neurodegenerative disorder that affects the central nervous system, causing rapid and progressive paralysis, muscle wasting, and respiratory failure^1^. Despite significant advances in understanding the disease’s underlying mechanisms, there is currently no definitive diagnostic test or biomarker available, making clinical criteria the primary means of diagnosis^2^. With a global prevalence of 4–6 cases per 100 000 individuals and an annual incidence of 1–2 cases per 100 000 individuals, ALS is a significant public health challenge^3^.

ALS is a complex disease with multiple genetic and environmental factors contributing to its development. While significant progress has been made in identifying the genetic causes of ALS, such as mutations in the SOD1, C9orf72, and FUS genes, the environmental factors associated with the disease remain poorly understood^4^. Exposure to toxins like lead and pesticides has been linked to an increased risk of developing ALS. Research on ALS has made significant progress in recent years, and emerging technologies have shown promising results in managing and treating the disease. These technologies include telemedicine, neuroimaging, genetic studies, artificial intelligence, and machine learning. However, despite these advances, managing ALS in Africa remains a significant challenge due to limited resources and infrastructure. This paper aims to provide an overview of the current status of ALS research in Africa, highlighting the progress made so far, the challenges faced, and future directions for research in the region.

## Methods

We conducted a comprehensive literature review to explore the current status and future directions of ALS research in Africa for this perspective article. Our search was carried out in the PubMed, Embase, and Web of Science databases, including articles published in the English language from January 2000 to March 2023. We used the following search terms: “ALS”, “amyotrophic lateral sclerosis”, “motor neuron disease”, “Africa”, “Sub-Saharan Africa”, “research”, and “epidemiology”. The articles were screened by title and abstract initially, and those that met the inclusion criteria were then reviewed in full text. Studies that reported on ALS research in Africa, including epidemiological studies, clinical trials, and reviews, were included. Studies conducted outside Africa or focused on other neurological conditions were excluded.

## Current state of ALS research in Africa

Despite the growing awareness of ALS as a significant global health concern, the available research data on motor neuron diseases, including ALS, in sub-Saharan Africa is limited. Emmanuel and Thomas reported that only eight genetic studies had been conducted in the region, mostly in South Africa, revealing a population-specific aetiology for ALS in African and non-African countries^5^. Despite further studies being conducted in several other sub-Saharan African nations, such as Nigeria, Senegal, Mali, and Ethiopia, the prevalence and incidence of ALS in Africa remain incomplete.

Some studies have suggested that the incidence of ALS may be lower among those born in sub-Saharan Africa than those born in Europe, North America, and Asia^6^. In a recent study conducted in South Africa, which is an ethnically diverse region, the incidence rate and presentation of ALS were investigated over 4 years, identifying 203 incident cases^2^. The study found significant variation in ALS incidence rates across different population groups, with the highest rates being observed in individuals of European ancestry and the lowest in individuals of African ancestry.

A hospital-based cohort study conducted across multiple African centres examined the sociodemographic and clinical features, treatments, and prognoses of ALS patients^7^. The study included 185 ALS patients from nine centres in eight African countries, revealing a male predominance and a median age of onset of 53 years. The study found that only 26.3% of patients received riluzole treatment, and the median survival time from diagnosis was 14 months. The subcontinental location and riluzole treatment both independently affected survival. The study provides crucial insights into the epidemiology of ALS in Africa and highlights the need for further research to enhance our understanding of the disease in this region.

Two separate studies shed light on the prevalence and risk factors associated with ALS in Africa. The first study involved a door-to-door survey in a Nigerian town with a population of 20 000 to evaluate the prevalence of neurological disorders, including ALS^8^. The study found that the prevalence ratio for ALS was 15 per 100 000, which was within the expected range for African countries. The study emphasised the need for further research and community-based interventions to improve the diagnosis, treatment, and management of neurological disorders in developing countries.

The second study, conducted in Senegal, aimed to identify risk factors for ALS^9^. The case-control study involved 23 ALS cases and 53 controls and was conducted between July 2015 and June 2017. The study found that living outside Dakar, being a farmer, exposure to pesticides, and chemical fertilizers were significantly associated with ALS. These findings highlight the potential contribution of environmental factors to ALS risk in Africa. The researchers suggest that a larger, multicentre study with more ALS patients would be necessary to further investigate additional risk factors and their causality in the African population.

The lack of population-based studies covering a significant portion of Asia, Latin America, and Africa makes it difficult to estimate the burden of motor neuron diseases worldwide. Nonetheless, with the rapid rise in living standards in nations with significant demographic impact, the burden of motor neuron diseases is anticipated to shift over the coming decades to Asia and Africa^10^. Therefore, it is crucial to increase research efforts in Africa to understand better the genetic and molecular causes of ALS and other motor neuron diseases. Such efforts could reveal therapeutically useful indicators for detecting, managing, and treating the disorder, leading to improved social care, public awareness of ALS, and the efficiency of professional diagnosis and therapy. Moreover, more studies are needed to determine the burden of motor neuron diseases in Africa, which could inform the development of appropriate strategies for disease prevention and management in the region.

In spite of the obstacles encountered by ALS research in Africa, several initiatives have been aimed at enhancing the situation. One such initiative is the establishment of ALS research networks and collaborations. AFRALS, the African Network for the Study of ALS, was established in 2017 to stimulate and coordinate ALS research in Africa^11^. AFRALS seeks to encourage partnerships among researchers, clinicians, and advocacy groups in the area to augment knowledge and resources for ALS research. Another initiative is the formation of ALS clinics and centres of excellence in several African countries^12^. These centres offer specialised care for ALS patients and function as research centres. For example, the MND/ALS clinic was established in Nigeria in 2015, and it has been providing clinical care and researching ALS ever since^13^. Likewise, the Neurology Department of the University of Cape Town in South Africa has an ALS clinic that provides clinical care and serves as a research centre for ALS^14^.

Moreover, international partnerships and collaborations have also been created to support ALS research in Africa. The ALS Association (ALSA), for instance, has provided funding and support for ALS research in Africa^15^. ALSA has also partnered with AFRALS to boost ALS research in the region. The European Union-funded Euro-MOTOR project also supports ALS research in Africa through collaborations with African researchers^16^. In addition, advocacy groups have also played a significant role in creating awareness and raising funds for ALS research in Africa. The ALS/MND Support Group of South Africa, for example, is a non-profit organisation that provides support for ALS patients and their families while also raising funds for research on ALS^17^.

## Challenges to ALS research in Africa

Despite the potential impact of ALS on the African population, ALS research in Africa faces significant challenges that hamper understanding and treating the disease. These challenges include limited funding, inadequate infrastructure, and a shortage of trained personnel. The lack of ALS-specific resources and facilities in many African countries further exacerbates these issues.

Insufficient funding poses a significant challenge to ALS research in Africa. As foreign institutions predominantly fund research into this debilitating neurodegenerative disorder, local funding sources are often scarce^18^. As a result, many researchers face financial constraints that limit the scope and quality of their studies. Such funding shortages also impact the dissemination of research findings as researchers often lack the resources necessary to publish in prestigious journals or present their work at high-profile conferences. Inadequate funding thus hinders the progress of ALS research in Africa. With limited resources, researchers struggle to procure essential equipment, recruit study participants, or conduct clinical trials, ultimately impeding efforts to understand and mitigate the disease’s effects. Furthermore, lack of funding can stifle efforts to disseminate research findings, preventing these insights from reaching the broadest possible audience.

Inadequate infrastructure represents another significant challenge to the advancement of ALS research in Africa. Insufficient availability of specialized facilities and equipment, including diagnostic tools and laboratory resources, impedes researchers’ capacity to study this neurodegenerative disorder comprehensively^19^. As such, the scope and quality of ALS research in Africa are often constrained^20^.

Moreover, the shortage of trained personnel, such as clinicians and researchers, exacerbates this problem, further impeding the progress of ALS research^21^. In Africa, the low number of ALS experts means that many patients are not diagnosed or treated appropriately, while few clinicians possess the necessary expertise to research the disease^22^. The implications of this dearth of specialized training are significant, impairing accurate diagnosis and monitoring of the disease, as well as the development of effective treatments. Furthermore, the shortage of specialized infrastructure and personnel limits collaboration between African and international researchers, restricting the exchange of knowledge and ideas^23^. This, in turn, limits the potential contribution of African research to the global understanding of ALS.

Inadequate ALS-specific resources and facilities in many African countries present a challenge to the advancement of ALS research in the region. With other prevalent diseases demanding more resources, the prioritisation of ALS research is often lower, leading to a scarcity of resources and infrastructure^24^. Consequently, the scope and quality of ALS research are restricted, further hampering the advancement of our understanding of the disease.

Furthermore, ethical considerations pose another significant obstacle to ALS research in Africa. Cultural and religious beliefs make it challenging to obtain informed consent for research participation, limiting the number of study participants. In addition, concerns about the exploitation of vulnerable populations or the use of genetic information for discriminatory purposes can further limit research studies’ participation. In this context, developing strategies to address these ethical considerations is crucial. Moreover, the use of animal models in ALS research presents ethical considerations that are not unique to Africa. However, in some African countries, cultural or religious beliefs prohibit animal research, impeding the development of effective therapies.

## Emerging technologies in ALS research in Africa

In recent years, emerging technologies have contributed to advancements in ALS research in Africa. One such technology is telemedicine, which is used to improve ALS patient care and research. The ALS Functional Rating Scale-Revised (ALSFRSr) is a widely-used tool for assessing ALS patient function, and telemedicine has allowed for remote administration of this tool^14^. Additionally, remote monitoring of ALS patients through telemedicine has enabled real-time tracking of disease progression and provided valuable data for research purposes.

Neuroimaging is another emerging technology that has proven useful in ALS research in Africa. MRI is commonly used to detect structural changes in the brain and spinal cord of ALS patients. PET has also been utilised to detect metabolic changes in the brain and spinal cord of ALS patients. Diffusion tensor imaging has been used to study the integrity of white matter tracts in the brain and spinal cord of ALS patients, which can provide insights into disease progression and potential therapeutic targets.

Genetic studies have also played an important role in advancing ALS research in Africa. Linkage analysis has been used to identify genetic mutations associated with familial forms of ALS, while whole-exome sequencing has been utilised to identify genetic variants associated with sporadic forms of ALS. Genome-wide association studies have also been conducted to identify genetic variants associated with ALS risk and disease progression.

These emerging technologies have provided valuable tools for ALS research in Africa and hold promise for further advancements in understanding the disease and developing effective treatments.

## Recommendation and future directions

In order to address the challenges faced by ALS research in Africa and improve patient outcomes, it is important to identify priorities for future research efforts. A comprehensive and systematic approach is needed to establish effective strategies for advancing ALS research in Africa.

One major priority for research on ALS in Africa is to conduct large-scale epidemiological studies to determine the prevalence, incidence, and risk factors for the disease in the region. Such studies are crucial for understanding the burden of the disease, informing prevention and management strategies, and identifying genetic and environmental factors that contribute to ALS development in the African population. These studies can provide essential insights into the distribution of ALS across different African countries and sub-regions, enabling the development of region-specific interventions. For example, a better understanding of the prevalence of ALS in different African populations can inform the allocation of resources for research and healthcare, ultimately improving outcomes for individuals with ALS. Furthermore, large-scale epidemiological studies can identify genetic mutations that may contribute to ALS development, providing insights into the underlying biology of the disease. Similarly, these studies can also identify environmental factors that may increase the risk of ALS, such as exposure to toxins or infectious agents. These findings can inform interventions to mitigate these risks and prevent the development of ALS.

Furthermore, developing culturally appropriate diagnostic and management tools is a critical priority for ALS research in Africa. With the diverse cultural and linguistic backgrounds of patients in the region, it is crucial to develop tools that are sensitive to these differences and can provide accurate diagnoses and effective treatments. Collaboration among researchers, clinicians, and patient advocacy groups is necessary to ensure that the needs of patients from diverse backgrounds are addressed. In addition to the development of culturally appropriate diagnostic and management tools, clinical trials of emerging treatments are also a key priority for ALS research in Africa. With the emergence of new treatments for ALS, there is a need for clinical trials to evaluate their safety and efficacy in African populations. This will help to identify the most promising treatments for ALS in the region and facilitate their uptake into clinical practice. Moreover, research into the pathogenesis of ALS in African populations could also inform the development of novel therapeutic approaches. Understanding the unique genetic and environmental factors that contribute to ALS in Africa can help to identify new targets for treatment and enable the development of region-specific interventions.

Collaboration among researchers, clinicians, and advocacy groups is essential for advancing ALS research in Africa. Building capacity through training programs, workshops, and mentoring initiatives will also be crucial in fostering collaborations and promoting the uptake of innovative research methods. Involving patient and caregiver groups in the research process can facilitate the development of research questions that are relevant to the needs of the ALS community in Africa.

Providing adequate funding and facilities for ALS research in Africa is also a critical component in advancing research efforts in the region. Limited funding from local sources is a significant challenge that impedes progress in ALS research. As such, foreign institutions often fund most research on ALS in Africa, leading to financial constraints that limit the scope and quality of investigations. To address this issue, governments, philanthropic organisations, and private institutions can increase their financial support for ALS research in Africa. This will allow for the expansion of research activities, including the conduct of large-scale epidemiological studies, clinical trials of emerging treatments, and research into the pathogenesis of ALS in African populations. In addition to funding, the provision of adequate facilities and equipment is crucial for ALS research in Africa. The lack of specialized facilities and diagnostic tools limits the capacity of researchers to study ALS, and the shortage of trained personnel hinders progress in research efforts. Building infrastructure and providing state-of-the-art facilities and equipment can help to address these challenges and facilitate the conduct of high-quality research.

Collaboration among researchers, clinicians, and patient advocacy groups is also essential for advancing ALS research in Africa. By working together, researchers can leverage each other’s expertise and resources, leading to a more comprehensive understanding of the disease and the development of effective diagnostic and management tools. The involvement of patient and caregiver groups in the research process can also help to ensure that research questions are relevant to the needs of the ALS community in Africa.

Moreover, building capacity through training programs, workshops, and mentoring initiatives can help to foster collaborations and promote the uptake of innovative research methods. This can enhance the skills and expertise of researchers and clinicians in the region, leading to the development of novel therapeutic approaches. With these efforts, significant strides can be made in improving outcomes for individuals with ALS in the region.

## Conclusion

ALS is a debilitating neurodegenerative disease that presents unique challenges to African researchers. Despite these challenges, there have been significant efforts towards improving the outcomes for patients in the region. In terms of the current status of ALS research in Africa, there are several key initiatives and emerging technologies that hold promise for advancing our understanding and management of the disease. These include the establishment of ALS research networks and collaborations, the development of specialised ALS clinics and centres of excellence, and the emergence of new technologies such as telemedicine, neuroimaging, and genetic studies. However, several challenges must be addressed to realise the potential of these initiatives and technologies fully. These challenges include limited funding and resources, a lack of collaboration and capacity building, and the need to integrate traditional and modern medicine in the care of ALS patients. Moving forward, it is important to prioritise ALS research in Africa and develop research strategies that are tailored to the unique challenges and opportunities of the region. Key priorities for future research efforts should include large-scale epidemiological studies, the development of culturally appropriate diagnostic and management tools, and clinical trials of emerging treatments.

## Ethical consent

NA.

## Consent

NA.

## Source of funding

None.

## Author contribution

Conceptualization: NA. Writing: all authors. Review: NA.

## Conflicts of interest disclosure

None.

## Research registration unique identifying number (UIN)

NA.

## Guarantor

Nicholas Aderinto.

## Data availability statement

NA.

## Provenance and peer review

Not commissioned, externally peer-reviewed.

## Assistance with the study

None.
